# Does sequential balloon expulsion test improve the screening of defecation disorders?

**DOI:** 10.1186/s12876-020-01490-x

**Published:** 2020-10-14

**Authors:** A. C. Caetano, D. Costa, R. Gonçalves, J. Correia-Pinto, C. Rolanda

**Affiliations:** 1 Department of Gastroenterology, Hospital of Braga, Sete Fontes – São Victor, 4710-243 Braga, Portugal; 2 Life and Health Sciences Research Institute (ICVS), School of Medicine, University of Minho, Braga, Portugal; 3 ICVS/3B’s-PT, Government Associate Laboratory, 4710-057 Braga, Guimarães, Portugal

**Keywords:** Constipation, Defecation disorders, Low-cost tools

## Abstract

**Background:**

A defecation disorder (DD) is a difficulty in evacuation documented by physiological exams. However, this physiological evaluation can be cumbersome, inaccessible and costly. Three “low-cost” tools to evaluate DD—a clinical DD score, the balloon expulsion test (BET) and a digital rectal examination (DRE) score were evaluated as separate or combined tests for DD screening.

**Methods:**

This prospective study occurred between January 2015 and March 2019 in the Gastroenterology Department of a tertiary hospital. Besides the gold standard physiological tests, constipated patients answered the clinical DD score and were evaluated by DRE and BET [standard and variable volume (VV)].

**Results:**

From 98 constipated patients, 35 (38.9%) were diagnosed with DD according to Rome IV criteria, mainly female (n = 30, 86%) with a median age of 60 years old. The clinical DD score revealed an AUC of 0.417 (SE = 0.07, *p* = 0.191). The DRE score displayed an AUC of 0.56 (SE = 0.063, *p* = 0.301). The standard BET displayed a sensitivity of 86%, specificity of 58%, positive predictive value (PPV) of 57% and negative predictive value (NPV) of 86%. The sequential VVBET followed by standard BET improved the BET performance regarding the evaluation of DD, with a sensitivity of 86%, specificity of 67%, PPV of 63% and NPV of 87%. The sequential BET had an OR 8.942, *p* > 0.001, CI 3.18–25.14, revealing to be the most significant predictor for DD screening.

**Conclusion:**

The sequential BET is a low cost, well-performing DD screening tool, appropriate to the Primary Care Setting.

## Background

A defecation disorder (DD) is defined as a difficulty in evacuation or emptying the rectum. DD may result from impaired anorectal function or rectal structural disturbances in patients with complaints of Chronic Constipation (CC) [1–3].

DD was recently defined by the Rome IV criteria, based on symptoms and objective physiological criteria—Table 1 [4, 5]. Therefore, the diagnosis of DD is established when a patient with functional chronic constipation (CC) or irritable bowel syndrome with constipation (IBS-C) has impaired evacuation as demonstrated by 2 of 3 types of tests—balloon expulsion test (BET); imaging (conventional defecography, dynamic ultrasound or dynamic magnetic resonance); anorectal manometry (ARM) or electromiography (EMG). This physiological evaluation is not always easily accessible, moreover it can be long and costly [3, 6, 7]. American studies report costs of healthcare utilization for CC as high as 500 dollars-patient-year while the exact impact of CC diagnostic assessment and treatment in Western Europe healthcare systems is unknown [8].

**Table 1:** Rome IV diagnostic criteria for functional defecation disorders

Rome IV Diagnostica criteria (a) for functional defecation disorders
1. The patient must satisfy diagnostic criteria for functional constipation and /or Irritable Bowel Syndrome with constipation
2. During repeated attempts to defecate, there must be features of impaired evacuation, as demonstrated by 2 of the 3 following 3 tests: a. Abnormal balloon expulsion test b. Abnormal anorectal evacuation pattern with manometry or anal surface electromyography c. Impaired rectal evacuation by imaging
(a) Criteria fulfilled for the last 3 months with symptoms onset at least 6 months before diagnosis

Thus, there is a subgroup of constipated patients—with DD—that can benefit from specific treatment behind laxatives, and we do not want them to miss proper treatment in consequence of the DD underdiagnosis. However, with the nowadays cost-effectiveness constraints, it may be impossible to perform the recommended physiological evaluation to all patients seeking a medical consultation for CC or IBS-C. A cheaper but satisfactory screening approach of DD that promotes an adequate selection of complementary tests and an earlier and adequate treatment seems ideal. Applying the creative concept of “low-cost” tools, we run an extensive review of the subject [9]. We found 3 potential “low-cost” tools—a DD clinical score, the BET and the digital rectal examination (DRE). Based on current evidence, it is not possible to know whether these “low-cost” strategies are useful in this setting [9].

Going into detail, no clinical score has emerged as a relevant diagnostic method in the diagram of DD and none was evaluated as a screening tool. Two specific DD scores (Altomare score and Renzi score) were validated to grade severity and value of treatment’s efficacy [10, 11]. Two important limitations prevented us from using Altomare score—it includes “time spent at the toilet” and “stool consistency”, items with a strong cultural influence and diet effect. The Renzi score is a 5-items score (Table 2) that assesses various complains of an abnormal evacuation and shows less cultural impact. The BET is a convenient procedure but described with inconsistent methodology especially regarding the volume used to inflate the balloon [9, 12–14]. Considering the physiological aspects of defecation, there remains doubts if a fixed low intrarectal volume is enough to trigger the desire to defecate or if higher variable volumes associated with the constant desire to evacuate can compensate for rectal hiposensitivity [2, 12]. The DRE is another low-cost tool that may sometimes be under or inappropriately used [15–17] and its value in DD was assessed in very few studies [9]. Only Tantiphlachiva proposed a DRE score to diagnose DD [18]. Therefore, there is a need for prospective studies with descriptive and consistent methodology to evaluate BET and DRE utility. In addition, it seems crucial an external validation of the few studies published so far [11, 12, 18]. Furthermore, no study in the literature evaluated a clinical DD score (or combination of its individual items) and these “low-cost” physiological tests—BET and DRE score. This effort seems relevant in the screening of DD in primary health care but also with potential utility in the diagnosis and assessment of treatment´s efficacy. This strategy may generate clear clinical and financial advantages [1, 2]. The aim of this study was to evaluate the performance of the “low-cost” tests in the screening of DD as separate or combined tools.

**Table 2:** Renzi clinical DEFECATION DISORDERS Score

Symptoms/score	0	1	2	3	4
Excessive straining	Never	Rarely	Sometimes	Usually	Always
Incomplete rectal evacuation	Never	Rarely	Sometimes	Usually	Always
Use of enemas/laxatives	Never	Rarely	Sometimes	Usually	Always
Vaginal/Perineal digital pressure	Never	Rarely	Sometimes	Usually	Always
Abdominal discomfort/pain	Never	Rarely	Sometimes	Usually	Always

Never, never; rarely, < 1/month; sometimes, < 1/week, ≥ 1/month; usually, < 1/day; ≥ 1/week; always, ≥ 1/day

## Methods

### Study design and subjects

This was a cross-sectional study. Consecutive patients with CC or IBS-C (Rome IV criteria), followed in the Department of Gastroenterology of Braga Hospital between January 2015 and March 2019 were prospectively proposed to the study protocol. The exclusion criteria were previous colonic and anorectal surgery, inflammatory bowel disease, colorectal cancer, anal cancer, other secondary causes of constipation, anorectal abnormalities that would influence symptoms of defecation detected by proctologic examination as anal fissure and haemorrhoids, incapacity to understand the study protocol.

During the clinical interview, the Renzi DD score was applied, followed by the DRE and the BET protocol, always by the same operator, as described in the next sections. The gold standard physiological tests (in our department anorectal manometry and defecation imaging) were subsequently scheduled and performed by another operator blinded to the study. A positive diagnosis of DD was considered according to 2 criteria of Rome IV, excluding information from the BET. Our constipated patients with no DD according with the current standards of diagnosis served as control group. Thus, patients with a positive BET plus a positive ARM or defecography were not included in DD group and were excluded from the control group (NoDD).

The Ethics Committee for Health of the Hospital of Braga approved the research protocol. Written informed consent was obtained from all participants. All data were collected anonymously and Portuguese regulations applicable to the management of personal data were followed at all times.

### Renzi DD score

A clinical DD score developed and validated by Renzi, as described in introduction and shown in Table 2, is divided in 5 items—excessive straining, incomplete rectal evacuation, use of enemas/laxative, vaginal/perineal digital pressure, abdominal discomfort/pain—that are scored from 0 to 4 points according to its frequency in everyday patients’ life. A final score ≥ 9 points was assumed as abnormal [11]. We validated the Portuguese version of the score [19] according to the Consensus based Standards for the selection of the Health Measurement Instruments (COSMIN) checklist [20] and following guidelines for cross-cultural validity [21]. The score was applied in a face to face interview. In this study, the score was also deconstructed and evaluated in diverse combinations of items.

### DRE technique

The general DRE was performed according to the technique described by Talley [15]. It was followed by the specific tests for pelvic floor dysfunction: the patient was requested to strain and try to push out the finger (to assess paradoxical external anal sphincter and puborectalis contraction), then the patients was asked regarding pain when pressing the posterior rectal wall (to assess puborectalis muscle tenderness) and then a hand was placed on the anterior abdominal wall of the patient while asking him/her to strain again (to assess if the patient is excessively contracting the abdominal wall). The DRE was performed always by the same examiner (the main investigator) who knew the patients previously from the outpatient consultation. Next, we applied the Tanthiplachiva score defining a positive diagnosis of DD when two of the following criteria were present: (1) paradoxical anal contraction or impaired anal relaxation, (2) impaired push effort, (3) absence of perineal descent [18].

### BET technique

With the patient lying in the left lateral position, an empty 4 cm long balloon covered with lubricating jelly and tied to a flexible catheter (external diameter, 6 mm) was placed in the rectum. The balloon was then filled with 50 ml of air through the catheter. The patient was asked to expel the balloon. A stop-watch was started and stopped when the patient expelled the device. The time taken for expelling the balloon was recorded. An abnormal BET (standard BET) was defined as inability to expel the balloon in less than 1 min. A second BET was performed following the same steps but with a variable volume of air—the volume of air associated with a constant desire to evacuate (vvBET).

### Statistical evaluation

Continuous data is presented as median and interquartile range. Normal distribution was checked using skewness and kurtosis. Comparisons among groups were carried out using the Chi-square test or Mann–Whitney test. The ROC curves were used to evaluate the performance of the continuous variables (clinical DD score and DRE score). Sensitivity and specificity were calculated for the abnormal clinical score, altered DRE, abnormal standard BET, abnormal vvBET and abnormal sequential BET. Candidate variables for inclusion in a prediction model were any significant (or borderline significant) variables at univariate analysis or variables whose inclusion was supported by the existing literature. Potential predictors were identified using backward stepwise selection. *p* values < 0.05 was defined for rejection of the null hypothesis. All the statistical analyses were conducted using the software SPSS 22.0 (IBM, USA).

## Results

From 98 patients with the clinical criteria of CC, 8 were excluded because there was not enough data to admit or exclude the DD or NoDD diagnosis. Thirty-five patients (38.9%) were diagnosed with DD, mainly female (n = 30, 86%) with a median age of 60 years old. The 55 constipated patients without criteria for DD (NoDD) were also mainly female (n = 51, 93%) but with a median age of 51 years old (*p* = 0.009). Table 3 describes demographic and clinical characteristics of the patients.

**Table 3:** Patients demographic and clinical characteristics

Characteristics	DD group	NoDD group	*p*
Age (years)	60	51	0.009
Gender (fem/male)	30/5	51/4	0.28
Total Renzi score	11	11	0.452
ARM rest pressure (mmHg)	62	63	0.792
ARM sustained pressure (mmHg)	101	105	0.805
ARM minimal sensitivity (ml)	97	96	0.991
ARM sustained sensitivity (ml)	203	192	0.668
ARM maximal sensitivity (ml)	242	221	0.302
Presence of anal defects	3	2	
Presence of structural abnormalities	18	12	

*ARM* anorectal manometry

### Renzi DD score

Regarding clinical complaints, excessive straining, was reported as usually or always by 64.7% of the DD patients and by 63.6% of the NoDD patients. Only 3 DD patients refer never perform excessive straining in evacuation and no patient of the NoDD group refer never perform excessive straining. Regarding incomplete rectal evacuation, 61.8% of the DD patients and 69% of the NoDD patients complain to feel it usually or always. Again only 2 patients in the DD group and 1 patient in the NoDD group describe never feel incomplete rectal evacuation. Regarding the use of enemas or laxatives, 48.5% of the DD patients refer to use it usually or always as well as 56.3% of the NoDD patients. Only 14.5% of the NoDD patients and 27.3% of the DD patients refer never use it. The vaginal/perineal digital pressure was usually or always used by 29.4% of the DD patients and by 16.3% of the NoDD patients; 52.9% of the DD patients and 43.9% of the NoDD patients refer never to require it. Abdominal discomfort or pain was felt usually or always by 34% of the DD patients and by 40% of the NoDD patients. Four patients in both groups never felt abdominal pain.

The clinical DD score was abnormal in 89% of the NoDD patients and in 64.7% of patients with DD (*p* = 0.04). Regarding each individualized item of the clinical DD score there were no significant differences between the groups (*p* > 0.05). The abnormal clinical DD score (score ≥ 9 points) displayed a sensitivity of 65%, specificity of 10%, positive predictive value (PPV) of 31% and negative predictive value (NPV) of 33%. It revealed an AUC of 0.417 (SE = 0.07, *p* = 0.191).

### DRE technique

Regarding the DRE, paradoxical anal contraction or impaired anal relaxation was identified in 31.4% of the DD patients and in 16.3% of the NoDD patients (*p* = 0.094). Impaired push effort was recognized in 34.3% of the DD patients and in 23.6% of the NoDD patients (*p* = 0.272). The absence of perineal descent was documented in 28.6% of the DD patients and in 27.3% of the NoDD patients (*p* = 0.893).

The DRE was abnormal in 18.2% of the NoDD patients and in 28.6% of DD patients (*p* = 0.248). The abnormal clinical DD score displayed a sensitivity of 29%, specificity of 82%, PPV of 50% and NPV of 64%. The DRE score displayed an AUC of 0.56 (SE = 0.063, *p* = 0.301).

### BET technique

The standard BET was abnormal in 41.8% of the NoDD patients and in 85.7% of DD patients (*p* < 0.001). Evaluating the vvBET, the median volume of the BET associated with a constant desire to evacuate was 133.2 ± 60.9 ml. The vvBET was abnormal in 32.7% of the NoDD patients and in 82.9% of DD patients (*p* < 0.001). The performance of the BET using fixed volume and variable volume was different in 6 patients—5 patients were capable of expelling the variable-volume balloon (normal vvBET) but not the fixed-volume balloon (abnormal standard BET) and one patient had the inverse performance.

The standard BET displayed a sensitivity of 86%, specificity of 58%, PPV of 57% and NPV of 86%. The vvBET alone showed a sensitivity of 83%, specificity of 67%, PPV of 62% and NPV of 86%. The sequential BET (vvBET followed by standard BET) improves the BET performance regarding evaluation of DD, with a sensitivity of 86%, specificity of 67%, PPV of 63% and NPV of 88%.

### Tool to screen DD

Table 4 displays the performing characteristics of the “low-cost” tools under evaluation.

**Table 4:** Performance of the low-cost tools

Tool /performance measurement	Sensitivity	Specificity	PPV	NPV	Accuracy
Abnormal (> 9 points) clinical DD score	65	10	31	33	31
Abnormal (≥ 2 points) Digital rectal examination	29	82	50	64	61
FV BET (standard)	86	58	57	86	69
VV BET	83	67	62	86	73
Sequential BET (VV » FV)	86	67	63	88	75

*DD* defecation disorder, *BET* balloon expulsion test, *VV* variable volume, *FV* fixed volume, *PPV* positive predictive value, *NPV* negative predictive value

At univariate analysis, only age (*p* = 0.022), standard BET (*p* < 0.001), vvBET (*p* < 0.001) and sequential BET (*p* < 0.001) were significant predictors of DD. Logistic regression demonstrated that sequential BET had an OR 8.942, *p* > 0.001, CI 3.18–25.14 and that the sequential BET standed out as the most significant predictor for screening DD.

## Discussion

Chronic constipation is one of the five most common gastrointestinal disorders. It consumes substantial health care resources due to the high prevalence and specificity of the diagnostic tests and treatments involved [3, 7]. In the current times, with financial cutbacks in healthcare, the judicious use of technology seems to be a relevant issue [7, 22]. Taking all these aspects in consideration, we selected 3 “low-cost” tools in order to understand their role in the screening of DD as potential tools to be used in a first approach, namely in Primary Care Setting.

As shown in Table 3, demographic and clinical characteristics of both DD patients and NoDD patients are identical except for age as DD patients are significantly older (60 vs 51 years old), that is also reported in other series and can be explained by the cumulative structural and physiological alterations in the pelvic floor of older women. (^1–3,6,23^).

The Renzi clinical DD score, in the original study, showed good discriminatory power to distinguish between patients and controls (sensitivity 92% and specificity 96%) and also variations in patients over time [11]. However, in our study population, the clinical score did not perform well as a screening tool (sensitivity 65% and specificity 10%). In fact, Renzi et al. validated their score specifically to grade defecation disorders and not as a diagnostic tool among constipated patients. Besides their patients’ sample was selected according to Rome III criteria and specific exclusion criteria—no irritable bowel syndrome and no slow transit constipation. That way it is difficult to reproduce their good results using their score as a screening tool. This goes in line with recent reviews that consider clinical scores useless for screening or diagnostic purposes in DD [24, 25]. One possible explanation is that patients, when asked about their symptoms, tend to exaggerate their complains when evoking them retrospectively. No item of the clinical DD score had a distinctive individualized performance, not even the most controversial item of the DD clinical score—“abdominal discomfort/pain”—pointing to the continuous spectrum of pain in the DD subgroup of these pathologies (IBS with constipation and CC).

The DRE score, similarly, did not perform well as a screening tool. Compared with the results presented by Tantiphlachiva in their sample of 209 patients (sensitivity of 75% and specificity of 87%), the DRE score had a poorer performance in our study population (sensitivity 29% and specificity 82%) [18]. The DRE is an operator-dependent technique. Although we tried to decrease this bias with the execution of the DRE always by the same operator, we still have to take into account the years of professional experience of the main investigator (5 years) compared with the Tantiphlachiva team. It would be interesting to evaluate the learning curve of the DRE technique. Another possible bias is the cultural barrier—it is a dynamic evaluation, and different populations may not consistently perform the same oral instructions.

The standard BET performance (evaluation of the ability to evacuate solid stool) is in accordance with the majority of the data presented in the literature [9]. Trying to discriminate the best performance of the BET, besides the standard BET, we evaluated the vvBET (evaluation of rectal sensory function, which can also disturb evacuation ability). The sequential BET, where vvBET is followed by standard BET, improved the BET performance regarding evaluation of DD. These results points to the importance of rectal filling and its conscious awareness for a correct BET, improving the BET capability as a screening tool. Our results validate Minguez et al. results [12]. We also shared their enthusiasm that simple tools can be easily performed in any examination room and can be incorporated in the preliminary evaluation of patients with CC. The sequential BET increases specificity, PPV and NPV to this tool. Increasing age can also add specificity to the BET sequence. So, the sequential BET proposed could become an interesting tool for screening constipated patients in the Primary Care setting.

This study has some limitations already pointed out—cultural barrier regarding the patients, years of experience regarding the investigators. The left lateral position to perform the BET can also be seen as a limitation as the sitting position is more physiological [26]. The sample size can also be seen as a limitation. In our defense, while both the clinical score and the DRE score did not reveal discriminative power to screen constipated patients, the standard BET had a similar performance to that reported by other series [9]. Besides, as we know, an algorithm or score always performs better in the validation population (reported in the original papers) and the consistency of the results when performing the external validation is often not achieved. Pursuing the refinement of the low-cost tools, the sequential BET seems the most suitable to potential use in the Primary Care Setting.

## Conclusion

The clinical DD score and the DRE did not reveal discriminative power to evaluate patients with DD. The BET stands as a good, reproducible and low-cost tool, that performs better when sequentially used with variable volume and fixed volume. Age can improve the BET specificity to exclude DD.

## Data Availability

The data is available from the corresponding author in digital format that can be sent to the journal.

## Abbreviations

AUC: Area under the curve
ARM: Anorectal manometry
BET: Balloon expulsion test
CC: Chronic constipation
DRE: Digital rectal examination
DD: Defecation disorder
EMG: Electromiography
IBS-C: Irritable bowel syndrome-subtype constipation
ROC: Receiver Operating Characteristic
VV: Variable volume
PPV: Positive predictive value
NPV: Negative predictive value
